# Supplementary figures and images for: Programmatic considerations and evidence gaps for chikungunya vaccine introduction in countries at risk of chikungunya outbreaks: Stakeholder analysis
Source: PLoS Negl Trop Dis. 2024 Apr 4;18(4):e0012075. doi: 10.1371/journal.pntd.0012075 (PMC11020901; doi:10.1371/journal.pntd.0012075)

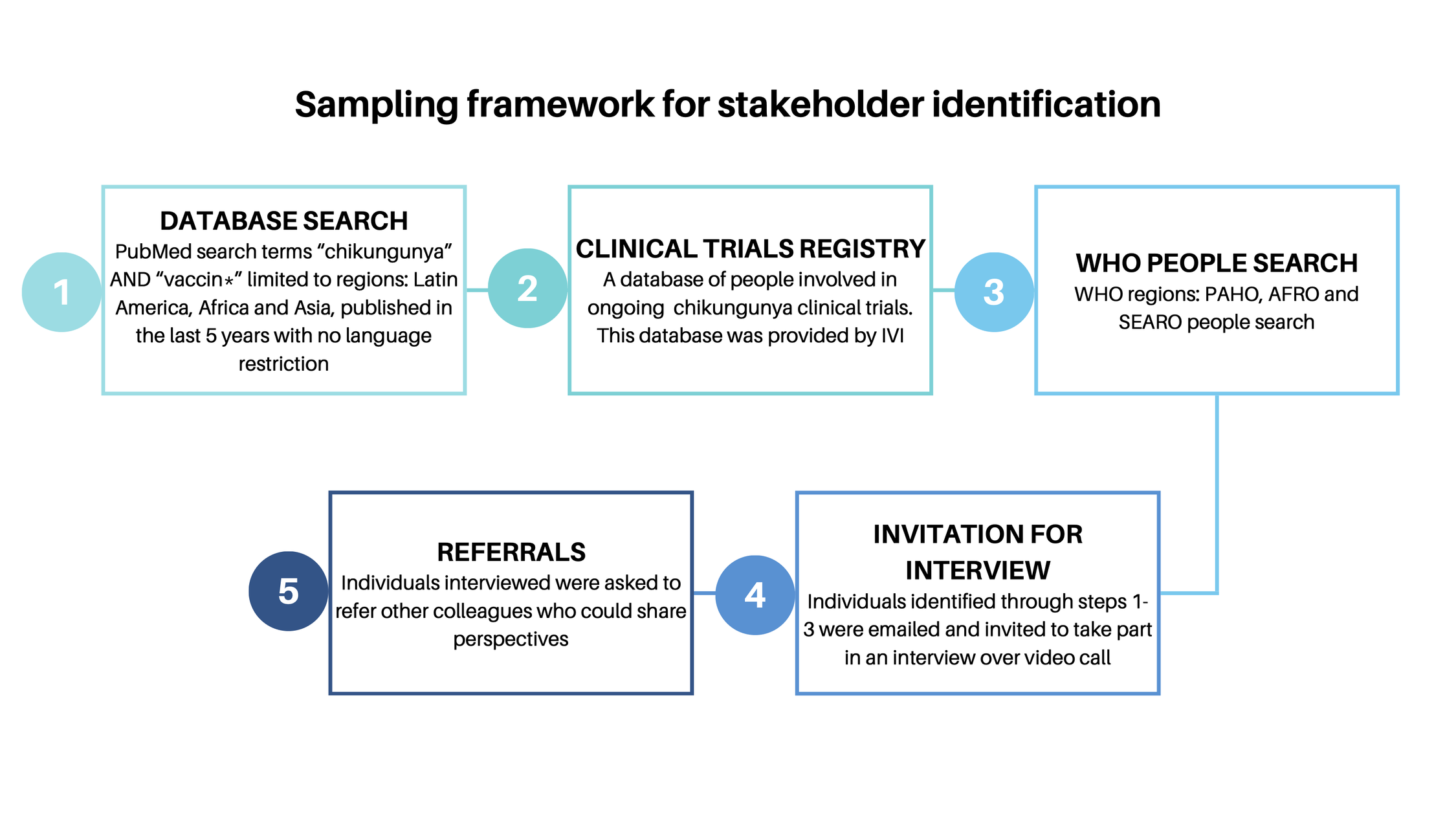

Supplement: S1 Fig — First, articles on chikungunya vaccination were identified from a PubMed search, limited to geographical regions with a high risk of chikungunya outbreaks. There was no language restriction on articles, but the publication date was limited to the last five years, in line with chikungunya vaccine development. Next, a database of individuals involved in chikungunya vaccine clinical trials were added to the list of stakeholders to contact. Third, WHO regional websites were used to identify individuals working on chikungunya in the AFRO, PAHO and SEARO regions. Individuals identified in steps 1–3 were then invited to partake in a stakeholder interview. If successful contact was made, during the interview stakeholders were invited to refer colleagues that would also be interested in sharing perspectives. (TIF) [file pntd.0012075.s001.tif]
