# Supplementary material for: Programmatic considerations and evidence gaps for chikungunya vaccine introduction in countries at risk of chikungunya outbreaks: Stakeholder analysis
Source: PLoS Negl Trop Dis. 2024 Apr 4;18(4):e0012075. doi: 10.1371/journal.pntd.0012075 (PMC11020901; doi:10.1371/journal.pntd.0012075)
Supplement: Attachment — Submitted filename: PNTD-D-23-01245R1_Response_to_Editor.docx [file pntd.0012075.s003.docx]

Manuscript ID: PNTD-D-23-01245R1

Dear Editor,

Following your email on 25 January 2024, requested revisions have been made and the responses noted below:

Dear Dr Auzenbergs,

Thank you for submitting your paper, "Programmatic considerations and evidence gaps for chikungunya vaccine introduction in countries at risk of chikungunya outbreaks: stakeholder analysis", to PLOS Neglected Tropical Diseases. We have checked over your submission and, before we can assign your manuscript to the Editor, we ask that you first please make the following modifications:

1) Please upload all main figures as separate Figure files in .tif or .eps format. For more information about how to convert and format your figure files please see our guidelines: 
[https://journals.plos.org/plosntds/s/figures](https://protect-eu.mimecast.com/s/oiOaCO8KBCr5MrrTPGYKJ?domain=journals.plos.org)

- *All main figures have now been uploaded as .tif files*

2) "Please ensure that the CRediT author contributions listed for every co-author are completed accurately and in full. At this stage, the following Authors require contributions: Megan Auzenbergs. Please ensure that the full contributions of each author are acknowledged in the ""Add/Edit/Remove Authors"" section of our submission form.

- *CRediT contributions for author Megan Auzenbergs have now been completed*

3) Please amend your detailed Financial Disclosure statement. This is published with the article, therefore should be completed in full sentences and contain the exact wording you wish to be published.
i). State what role the funders took in the study. If the funders had no role in your study, please state: “The funders had no role in study design, data collection and analysis, decision to publish, or preparation of the manuscript.”
ii). If any authors received a salary from any of your funders, please state which authors and which funders.

- *The funders had no role in this study. Accordingly, statement (i) has now been included in the financial disclosure section of the manuscript lines 519-524:*
  - *“This project was funded by the International Vaccine Institute. HK is supported by the Vaccine Impact Modelling Consortium. OJB was supported by a UK Medical Research Council Career Development Award (MR/V031112/1). KA is supported by the Vaccine Impact Modelling Consortium (INV-034281) and the Japan Agency for Medical Research and Development (JP223fa627004). The funders had no role in study design, data collection and analysis, decision to publish, or preparation of the manuscript.”*

4) Since your data is not available for proprietary reasons, please include the contact information for the third party organization that should be contacted should other researchers want to request access to this data and please include the full citation of where the data can be found. We also request that you verify with us via email that any researcher will be able to obtain the data set in the same manner that the you have obtained it. If you feel you are unwilling or unable to adhere to this policy, please explain your reasons by return email and your exemption request will be escalated to the editor for approval. Your exemption request will be handled independently and will not hold up the peer review process, but will need to be resolved should your manuscript be accepted for publication. One of the Editorial team will be in touch if they require more information.

- *Upon reasonable request, survey data will be shared anonymously without identifying the stakeholder names and their affiliated organisations. Reasonable requests should be made to the corresponding author, Megan Auzenbergs at megan.auzenbergs@lshtm.ac.uk*

5) Please ensure that the Author List in your manuscript file matches the Author List in the online submission form exactly. Authors should be listed in the same order, and the order of individual first and last author names must be identical in both locations.

- The order of authors listed on the paper now matches the online submission portal

6) Please provide an Author Summary. This should appear in your manuscript between the Abstract (if applicable) and the Introduction, and should be 150–200 words long. The aim should be to make your findings accessible to a wide audience that includes both scientists and non-scientists. Sample summaries can be found on our website

- *An author summary section has now been written; it is on lines 30-41 of the main text document, following the abstract*

7) We have noticed that you have uploaded Supporting Information file, but you have not included a list of legends. Please add a full list of legends for your Supporting Information files after the references list.

- *A list of legends for the Supporting Information files have been listed after the references list in the main text document, lines 645-658.*

8) Thank you for stating"Ethical approval for this project was received from the London School of Hygiene and Tropical Medicine in January 2023, project reference number 28292".
Please include a statement that formal consent was obtained (must state whether verbal/written) OR the reason consent was not obtained (e.g. anonymity). NOTE: If child participants, the statement must declare that formal consent was obtained from the parent/guardian/head of household"

- *Written consent was obtained from all stakeholders before interviews took place. This has now been explicitly stated in the manuscript on lines 161-162.*

9) The Uploaded Manuscript (REVISED_CLEAN_TEXT_ONLY.docx) file contains highlights (Line n0 425,432,436,486,488). Please provide clean version of your manuscript from your latest manuscript version.

- *The lines in question do not show any highlights on the approved pdf generated by the journal’s submission portal. We have double the final text that has been re-submitted and do not find any remaining highlights.*

Once you have made the necessary changes in Editorial Manager, please re-approve the submission by following the same steps as before.

Please resolve these issues and resubmit your manuscript within 30 days. If you need more time, please email the journal office at [plosntds@plos.org](mailto:plosntds@plos.org). We are happy to grant extensions of up to one month past this due date. If we do not hear from you within 30 days, we will withdraw your manuscript on your behalf.

If you have any questions or require assistance, please don't hesitate to get in touch. To help us minimize inbox volumes, we kindly ask that you not reply to this email simply to confirm you have resubmitted your revisions.

Kind regards,

Nithya Venkatesan
PLOS Neglected Tropical Diseases
[plosntds@plos.org](mailto:plosntds@plos.org)
